# Contourlet Textual Features: Improving the Diagnosis of Solitary Pulmonary Nodules in Two Dimensional CT Images

**DOI:** 10.1371/journal.pone.0108465

**Published:** 2014-09-24

**Authors:** Jingjing Wang, Tao Sun, Ni Gao, Desmond Dev Menon, Yanxia Luo, Qi Gao, Xia Li, Wei Wang, Huiping Zhu, Pingxin Lv, Zhigang Liang, Lixin Tao, Xiangtong Liu, Xiuhua Guo

**Affiliations:** 1 School of Public Health, Capital Medical University, Beijing, China; 2 Beijing Municipal Key Laboratory of Clinical Epidemiology, Beijing, China; 3 School of Medical Sciences, Edith Cowan University, Perth, Australia; 4 School of Exercise and Health Sciences, Edith Cowan University, Perth, Australia; 5 Department of Epidemiology & Public Health, University College Cork, Cork, Ireland; 6 Department of Radiology, Beijing Chest Hospital, Capital Medical University, Beijing, China; 7 Department of Radiology, Xuanwu Hospital, Capital Medical University, Beijing, China; University of Campinas, Brazil

## Abstract

**Objective:**

To determine the value of contourlet textural features obtained from solitary pulmonary nodules in two dimensional CT images used in diagnoses of lung cancer.

**Materials and Methods:**

A total of 6,299 CT images were acquired from 336 patients, with 1,454 benign pulmonary nodule images from 84 patients (50 male, 34 female) and 4,845 malignant from 252 patients (150 male, 102 female). Further to this, nineteen patient information categories, which included seven demographic parameters and twelve morphological features, were also collected. A contourlet was used to extract fourteen types of textural features. These were then used to establish three support vector machine models. One comprised a database constructed of nineteen collected patient information categories, another included contourlet textural features and the third one contained both sets of information. Ten-fold cross-validation was used to evaluate the diagnosis results for the three databases, with sensitivity, specificity, accuracy, the area under the curve (AUC), precision, Youden index, and *F*-measure were used as the assessment criteria. In addition, the synthetic minority over-sampling technique (SMOTE) was used to preprocess the unbalanced data.

**Results:**

Using a database containing textural features and patient information, sensitivity, specificity, accuracy, AUC, precision, Youden index, and *F*-measure were: 0.95, 0.71, 0.89, 0.89, 0.92, 0.66, and 0.93 respectively. These results were higher than results derived using the database without textural features (0.82, 0.47, 0.74, 0.67, 0.84, 0.29, and 0.83 respectively) as well as the database comprising only textural features (0.81, 0.64, 0.67, 0.72, 0.88, 0.44, and 0.85 respectively). Using the SMOTE as a pre-processing procedure, new balanced database generated, including observations of 5,816 benign ROIs and 5,815 malignant ROIs, and accuracy was 0.93.

**Conclusion:**

Our results indicate that the combined contourlet textural features of solitary pulmonary nodules in CT images with patient profile information could potentially improve the diagnosis of lung cancer.

## Introduction

Lung cancer is a disease characterized by uncontrolled cell division in the tissues of the lung, and is the most common cause of cancer-related death in men and women worldwide [1]. The presence of lung cancer often appears as a solitary pulmonary nodule (SPN) as well as other lung lesions. An SPN is a single, spherical, well-circumscribed, radiographically opaque object that measures up to 3 cm in diameter and is completely surrounded by aerated lung tissue [2]. The definitive diagnosis of lung cancer is based on histological examination, which is usually performed by bronchoscopy or computed tomography- (CT)-guidance. Individuals who show the presence of these observations often have a low five-year survival rate (about 15%) [3]. CT technology, a useful computer aided diagnosis tool used in lung cancer detection, is used to screen and forecast patients with solitary pulmonary nodules (SPNs). With the low-dose CT screening, a 20% reduction of mortality was shown in lung cancer cases [4].

Morphological characteristics, such as: nodule size, density, and margins shown in CT slices, coupled with demographic characteristics, such as: gender, age, and smoking history, amongst other characteristics are used to differentiate between benign and malignant SPNs [5]. There are several studies that have evaluated the use of different combinations of these characteristics in prediction models in attempts to increase the accuracy of spotting potential malignant nodules. Using some of these features, Gould *et al*. [6] established a logistic regression based clinical prediction model to estimate the pre-test probability of malignancy in patients with SPNs, with a model accuracy of 0.79 in its area under the curve of receiver operating characteristic. Li *et al*. [7] also established a mathematical model to predict malignancy of SPNs, with a higher accuracy rate (0.888) compared to the Mayo model and VA group model. Although these studies obtained good results, textural features such as entropy, correlation, energy, homogeneity, etc. which are considered vital components were omitted from their research. Way *et al*. [8] used a fully automated system to extract image features to differentiate malignant and benign lung nodules on CT scans, in combination with morphological and demographic features. Zhu *et al*. [9] on the other hand used 25 features selected from 67 features, extracted by a feature extraction procedure to establish SVM-based classifier. Research by Wu *et al*. [10] used two GLCM based textural features and two radiological features as determined by non-linear regression to build a back propagation artificial neural network diagnosis model. Sun *et al*. [2] used a dataset including 476 textural features extracted by curvelet, three demographic parameters and nine morphological features to establish a support vector machine (SVM) prediction model.

The choice of methods used to extract textural features, have also established their importance in this process. Dettor *et al*. [11] and Meselhy *et al*. [12] found that the curvelet transform yielded better results in image processing than previous methods. Inspired by curvelet, Do and Vetterli [13] developed the contourlet transform, which allows for differences and flexibility in the number of directions permitted at each scale compared to other multiscale directional systems. In this study, a contourlet was used to extract textural features as a trial and SVM, which was more suitable for CT texture analysis than the other six models in previous studies [14], used to predict lung cancer. The aim of this study was to determine the value of contourlet textural features by comparing the diagnostic effect of datasets combining textural features and patient information, with datasets containing only patient information or textural features.

## Methods

### Ethics Statement

This study was performed with ethics approval (Ethics Committee of Xuanwu Hospital, Capital Medical University, Approval Document NO. [2011] 01). Written consent was given by the patients.

### Survey of patient information

The data were obtained from Chaoyang Hospital and Beijing Chest Hospital in Beijing, which is part of a cross-sectional study established in 2009 [14]. Seven demographic parameters (age, gender, smoking habits, tuberculosis history, dust history, genetic disease and tumour history) were obtained from medical histories of patients. In addition, twelve morphological features of the pulmonary nodules (calcification, cavitation, density, ground-glass, lobulation, lymph node status, margin, vacuoles, pleural indentation, pleural fluid, diameter, and substantial changes) reported by experienced radiologists for the patients were also included in this study. The distributions of age and diameter were continuous. In gender, 1 represented female, 0 represented male while for the other variables, 1 represented yes, and 0 represented no. The patient inclusion/exclusions decision was based on the results of the final diagnoses where the final diagnosis of malignant cases was confirmed by an operation or biopsy, while benign cases were determined either pathologically or after a 2-year minimum follow-up by patients. Morphological features in two dimensional CT images were obtained by eight radiologists and conflicts in the final interpretation of the CT images were resolved through consensus discussion. All of the nineteen variables were utilized as patient information data categories.

### Feature extraction strategies

A total of 6,299 regions of interest (ROIs) were acquired from 336 patients, with 1,454 benign ROIs from 84 patients (50 male, 34 female) and 4,845 malignant ROIs from 252 patients (150 male, 102 female). These details are listed in Table 1.

**Table 1:** Description of the data.

Diagnosis		Cases	(%)	ROIs	(%)
Benign		84	100.0	1454	100.0
	Tuberculosis	28	33.3	496	34.1
	Inflammatory pseudotumor	15	17.9	265	18.2
	Hamartoma	20	23.8	367	25.2
	Pulmonary interstitialedema	2	2.4	34	2.3
	Sclerosing hemangioma	12	14.3	189	13.0
	Clear cell tumor	2	2.4	31	2.1
	Chondroma	5	6.0	72	5.0
Malignant		252	100.0	4845	100.0
	Adenocarcinoma	183	72.6	3443	71.1
	Squamous cell carcinoma	45	17.9	887	18.3
	Adenosquamous carcinoma	18	7.1	379	7.8
	Malignant carcinoid tumor	6	2.4	136	2.8

CT scans were obtained using a 64-slice helical CT scanner (GE/Light speed ultra System CT99, USA) with a tube voltage of 120 kV and a current of 200 mA [10]. The reconstruction interval and reconstruction thickness for routine scanning were 0.625 mm. The kernel was a B31f/B70 type and the data were reconstructed using a 512×512 matrix [15].

**Figure 1:**
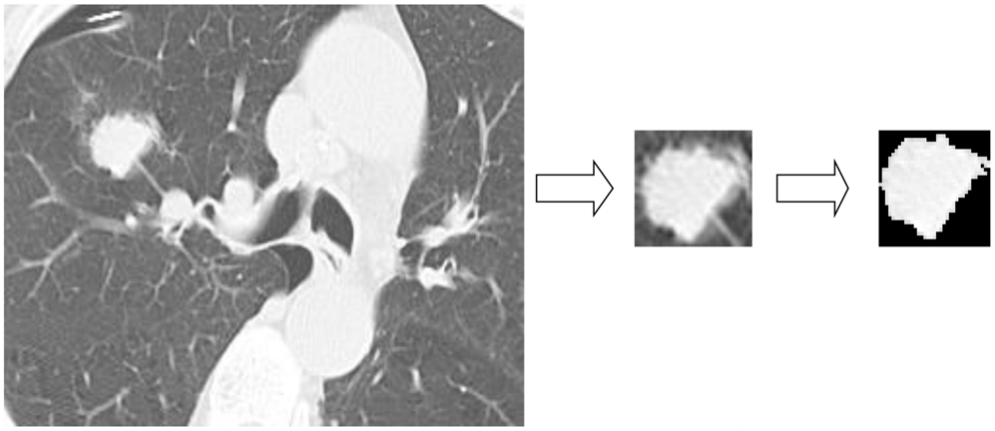
Image segmentation using gray level threshold algorithm.

All of the pulmonary nodules in CT images were segmented manually to obtain a region of interest (ROI) and the textural features were extracted by contourlet from each ROI. Figure 1 shows an example of image segmentation. The ROI was obtained from CT image by a rectangular box, which covered the whole ROI at the smallest area. Subsequently Region Grow Algorithm, a popular tool for image segmentation, was used to remove any background pixels, such as muscle and blood vessels. Using a validated contourlet transform method [13], a filter bank structure capable of proficiently working with piecewise smooth images with smooth contours. Fourteen kinds of textural features were extracted. These included entropy, mean, correlation, energy, homogeneity, standard deviation, maximum probability, inverse difference moment, cluster tendency, inertia, sum-mean, difference-mean, sum-entropy, and difference-entropy [16]. The contourlet transform process included two steps: a Laplacian pyramid (LP) and a directional filter bank (DFB). The LP was first used to capture point discontinuities and the DFB subsequently used to link point discontinuities into linear structures [17]. In this study, 48 sub-bands were chosen, resulting in 672 textural features calculated. The differences of textual features between benign and malignant group were subsequently analyzed. The comparative evaluation of each kind of textural features and the results are shown in Table 2. The least areas under the curve (AUC) obtained from cluster tendency, mean, sum-mean were 0.53, 0.53 and 0.52 respectively with *P* values not smaller than 0.05, while *P* values of other textural features were smaller than 0.05. All the textual features between benign and malignant group were analyzed to determine their differences. Table S1 displays the ones with *P* value smaller than 7.4e-5.

**Table 2:** The performance of each kind of textural features.

Textural features	Sensitivity	Specificity	Youden	AUC	Accuracy	Precision	F
Correlation	0.69	0.55	0.24	0.63	0.65	0.82	0.75
Cluster tendency	0.81	0.29	0.10	0.53*	0.68	0.77	0.79
Difference-entropy	0.63	0.52	0.15	0.58	0.60	0.80	0.70
Difference-mean	0.67	0.48	0.15	0.57	0.63	0.79	0.73
Energy	0.81	0.38	0.19	0.60	0.70	0.80	0.80
Entropy	0.66	0.55	0.21	0.62	0.63	0.81	0.73
Homogeneity	0.73	0.45	0.18	0.59	0.66	0.80	0.76
IDM	0.66	0.54	0.19	0.60	0.63	0.81	0.73
Inertia	0.67	0.57	0.24	0.63	0.65	0.82	0.74
Mean	0.68	0.43	0.11	0.53*	0.62	0.78	0.73
MP	0.68	0.54	0.21	0.61	0.64	0.81	0.74
SD	0.53	0.62	0.15	0.59	0.55	0.81	0.64
Sum-mean	0.82	0.29	0.10	0.52*	0.68	0.77	0.80
Sum-entropy	0.58	0.62	0.19	0.63	0.59	0.82	0.68

Abbreviation used: AUC, the least area under the curve; IDM, Inverse difference moment; MP, Maximum probability; SD, Standard deviation; F, F_measure;

*P>0.05.

### Data analysis

In our study, a support vector machine (SVM), which is a popular machine learning technique, established by recent studies in this field, was used in pattern recognition and classification in various research fields [18]–[20]. Developed for binary (two-class) classification problems, it can efficiently perform a non-linear classification using what is called a kernel function, implicitly mapping inputs into high-dimensional feature spaces. In this study, the Gaussian radial basis function kernel (Eq. 1) was chosen as the kernel function and 10-fold cross validation was used to access the datasets with and without, contourlet-based textural features.
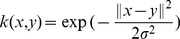
(1)


The original training data included images of 1,454 benign ROIs and 4,845 malignant ROIs, with a ratio of malignant to benign cases of 3, which were not balanced. The synthetic minority over-sampling technique (SMOTE) was used to preprocess the data, which was one of over-sampling method [21]. The purpose of applying this method is to generate a balanced dataset by generating new examples of minority class using the nearest k (which is set to 5 in SMOTE) neighbors of these cases while under-sampling majority class examples.

### Assessment criteria

Five indicators were calculated to evaluate the results using three datasets, including sensitivity, specificity, accuracy, Yonden index and precision. Additionally, the area under the curve (AUC) was calculated to establish the received operation characteristic (ROC), and malignant rate (Eq. 2) was used as variable to draw an ROC curve.
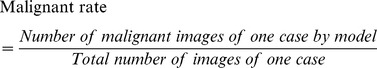
(2)


The F-measure (F), typically used in machine learning, was also calculated to measure the quality of the binary classifications as expressed by
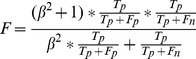
(3)where 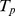 refers to the number of malignant nodules correctly classified as malignant and 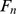 indicates the number of malignant nodules wrongly classified as benign; 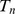 represents the number of benign nodules correctly classified as benign and 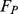 indicates the number of benign nodules wrongly classified as malignant. The *F*-measure is a measure of the accuracy of a test and when *β* is selected as 1, it can be interpreted as a weighted average of sensitivity and precision, with its best value occurring at 1 and worst at 0.

## Results

**Figure 2:**
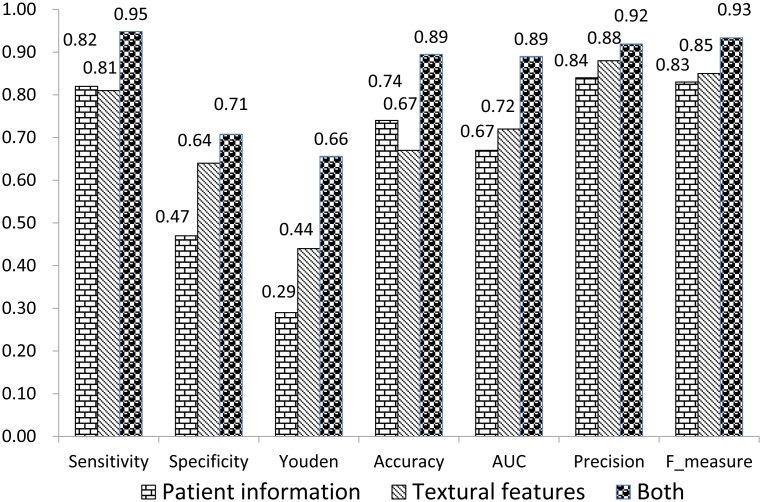
Results of three datasets run through the support vector machine.

The distribution of demographic parameters between benign and malignant cases is shown in Table 3, with the distribution of twelve morphological features displayed in Table 4. Table 5 shows comparative evaluations of textural features in different nodule sizes, which are 7 to 10 millimeters (group A, 26 cases), 11 to 20 millimeters (group B, 129 cases) and 21 to 30 millimeters (group C, 181 cases). As AUC obtained from group A was 0.70 with a *P* value of 0.073 which was not smaller than 0.05, we combined group A and group B, with its results also shown in Table 5. Three datasets, one comprising the seven demographic parameters and twelve morphological features, the second with only contourlet-based textural features, and the third one containing both, were used as input data to establish three separate SVM prediction models, respectively. Through ten-fold cross validation, seven indicators, including sensitivity, specificity, accuracy, AUC, precision, Youden index, and *F*-measure, were calculated to compare the three datasets, which were shown in Figure 2. Using the SMOTE as a pre-processing procedure, new data including the contourlet-based textural features, seven demographic parameters and twelve morphological features were generated, resulting in final data containing observations of 5,816 benign ROIs and 5,815 malignant ROIs. Accuracy based on ten-fold cross validation for balanced data were 93.3%.

**Table 3:** Distribution of seven demographic parameters: benign and malignant cases.

Variables	Benign	Malignant	Statistic	P
Gender				
N (missing)	84 (0)	252 (0)	0.00	1.0000
Female (%)	34 (40.48)	102 (40.48)		
Male (%)	50 (59.52)	150 (59.52)		
Smoking habits				
N (missing)	84 (0)	252 (0)	26.78	<0.0001
Yes (%)	24 (28.57)	154 (61.11)		
No (%)	60 (71.43)	98 (38.89)		
Age				
N (missing)	84 (0)	252 (0)	3.45	0.0006
Mean (std)	54.10 (13.57)	59.90 (12.68)		
Median (Q1, Q3)	57 (46.5,63)	61 (53,69.5)		
Min∼Max	21∼80	25∼83		
Tuberculosis history				
N (missing)	84 (0)	252 (0)	1.13	0.2869
Yes (%)	6 (7.14)	15 (5.95)		
No (%)	78 (92.86)	237 (94.05)		
Tumor hisory				
N (missing)	84 (0)	252 (0)	1.13	0.2869
Yes (%)	3 (3.57)	17 (96.75)		
No (%)	81 (96.43)	235 (93.25)		
Genetic disease				
N (missing)	84 (0)	252 (0)	-	0.5760
Yes (%)	0 (0)	3 (1.19)		
No (%)	84 (0)	249 (98.81)		
Dust history				
N (missing)	84 (0)	252 (0)	0.05	0.8255
Yes (%)	1 (1.19)	6 (2.38)		
No (%)	83 (98.81)	246 (97.62)		

**Table 4:** Distribution of twelve morphological features: benign and malignant cases.

Variables	Benign	Malignant	Statistic	P
Lymphadenectasis				
N (Missing)	84 (0)	252 (0)	10.32	0.0013
No (%)	73 (86.90)	174 (69.05)		
Yes (%)	11 (13.10)	78 (30.95)		
Uniform density				
N (Missing)	84 (0)	252 (0)	0.04	0.8455
Yes (%)	31 (36.90)	96 (38.10)		
No (%)	53 (63.10)	156 (61.90)		
Substantial changes				
N (Missing)	84 (0)	252 (0)	0.04	0.8345
No (%)	9 (10.71)	25 (9.92)		
Yes (%)	75 (89.29)	227 (90.08)		
Ground-glass				
N (Missing)	84 (0)	252 (0)	0.01	0.9045
No (%)	78 (92.86)	233 (92.46)		
Yes (%)	6 (7.14)	19 (7.54)		
Spiculation				
N (Missing)	84 (0)	252 (0)	0.05	0.8304
No (%)	23 (27.38)	66 (26.19)		
Yes (%)	61 (72.62)	186 (73.81)		
Lobulation				
N (Missing)	84 (0)	252 (0)	0.29	0.5929
No (%)	20 (23.81)	53 (21.03)		
Yes (%)	64 (76.19)	199 (78.97)		
Vacuoles				
N (Missing)	84 (0)	252 (0)	2.38	0.1227
No (%)	66 (78.57)	216 (85.71)		
Yes (%)	18 (21.43)	36 (14.29)		
Calcification				
N (Missing)	84 (0)	252 (0)	0.52	0.4704
No (%)	77 (91.67)	224 (88.89)		
Yes (%)	7 (8.33)	28 (11.11)		
Cavitation				
N (Missing)	84 (0)	252 (0)	1.71	0.1909
No (%)	78 (92.86)	221 (87.70)		
Yes (%)	6 (7.14)	31 (12.30)		
Pleural indentation				
N (Missing)	84 (0)	252 (0)	0.45	0.5021
No (%)	54 (64.29)	172 (68.25)		
Yes (%)	30 (35.71)	80 (31.75)		
Pleural fluid				
N (Missing)	84 (0)	252 (0)	0.01	0.9157
No (%)	76 (90.48)	227 (90.08)		
Yes (%)	8 (9.52)	25 (9.92)		
Diameter				
N (Missing)	84 (0)	252 (0)	4.50	<0.0001
Mean (Std)	1.80 (0.68)	2.22 (0.73)		
Median (Q1∼Q3)	1.8 (1.2∼2.3)	2.3 (1.7∼2.7)		

**Table 5:** The performance of classifier in different nodule size.

Groups	Sensitivity	Specificity	Youden	Accuracy	AUC	Precision	F_measure
A	0.77	0.62	0.38	0.69	0.70*	0.67	0.71
B	0.92	0.65	0.57	0.83	0.73	0.84	0.88
C	0.93	0.43	0.36	0.86	0.65	0.90	0.92
A+B	0.92	0.66	0.58	0.83	0.74	0.83	0.87

Abbreviation used: AUC, the least area under the curve; A, nodules within the 7 to 10 millimeters; B, nodules within the 11 to 20 millimeters; C, nodules within the 21 to 30 millimeters;

*P>0.05.

## Discussion

Lung cancer, the most common cancer-related death worldwide, accounted for 28% and 26% of all cancer deaths among men and women, respectively in the United States [22], [23] and has also been ranked first amongst causes of mortality involving malignant neoplasms in China [24]. This study is aimed at using textural features extracted by contourlet, incorporated with patient information, with the intention of establishing an SVM model, that will better assist in discerning between malignant and benign pulmonary nodules in the diagnosis of lung cancer as opposed to utilizing demographic and morphological indicators or nodule textural features alone. When combined with textural features, the accuracy rate improved from 0.74 to 0.89, with sensitivity and specificity showing a 0.13% and 0.24% improvement respectively. A meta-analysis [25] showed a pooled sensitivity of 0.57 (95% confidence interval, 0.49 to 0.66) for CT scanning in lung cancer. The sensitivity in this study was also improved after combining with the textural features (from 0.82 to 0.95), along with other assessment criteria, including *F*-measure which is used in machine learning. In addition to comparing results between datasets constructed from only textual features and one containing both, the study also reinforced the need for greater patient information to improve the diagnosis of lung cancer.

The top 4 textural features that attained better Youden index, were inertia, correlation, maximum probability and entropy, while the top 4 acquainted with sensitivity were sum-mean, cluster tendency, energy and homogeneity, and the top 4 for specificity were sum-entropy, standard deviation, inertia and correlation. In our study, we were unable to identify one particular textural feature acquainted with both better sensitivity as well as better specificity that could be utilized in combination to produce an overall relatively higher sensitivity and specificity.

A contourlet transform is a multi-directional, multi-scale transform used in medical imaging [26], [27]. It uses elongated basis functions with different aspects to capture smooth contours in images. For images in DICOM format, the contourlet data provided the algorithm with more efficiency and robustness against the effect of noise compared to other transforms [28], and as such this was our justification for using it to extract the textural features required for this study. To determine which size of nodules were more suitable for the 48 subbands of textural features extracted by contourlet, comparative evaluations of textural features in different nodule sizes were added. These included nodules within the 7 to 10 millimeters group, those within the 11 to 20 millimeters group and nodules that fell within the 21 to 30 millimeters groups. Nodules within the 11 to 20 millimeters group achieved relatively better results as indicated by their AUC values while AUC obtained from 7 to 10 millimeters group was 0.70 with P value of 0.073 which was not smaller than 0.05, we combined to 7 to 20 millimeters group, with performance of classifier did not change so much with 11 to 20 millimeters group. One reason may be that the 48 subbands of textural features extracted by contourlet were more suitable for pulmonary nodules smaller than 20 millimeters, especially 11 to 20 millimeters. Another may be that the cases in 7 to 10 millimeters group were not enough and 21 to 30 millimeters group had the highest malignant rate (28 benign cases vs 153 malignant cases). We will try to collect more data for further exploration, especially benign cases and cases in smaller size. In addition, SVM, has been considered to be a good algorithm for classification in various research fields, such as: clinical form analysis [29], cancer diagnosis [30]–[32], subtle gesture recognition [33], *etc*. and was therefore utilised to differentiate between lung cancer and benign lesions.

In this study, the accuracy rate in this study was not shown to be better than some previous studies [2], [10], [14] reported. The purpose of this study was to determine the value of textural features by comparing the predictive effect of three databases, whose input data were raw data without pre-processing. One of the limiting factors was that data required for such a study can only rely on cases obtained from hospitals. As such, there is a difficulty in obtaining a comparable number of benign and malignant cases as there is a propensity for malignant cases to outnumber benign ones. The malignant cases from our study were approximately three times as frequent as benign ones. This could have contributed to the poor specificity obtained. In this study, the SMOTE, an over-sampling method, was used as the pre-processing procedure to balance the data. The original training data was balanced with a ratio of malignant to benign cases of 1∶1 and the classification performance (accuracy) of the prediction model improved from 0.89 to 0.93. Thus, the SMOTE is a useful method to account for unbalanced data and can improve the capability of the models. The reasons why it performed better may be that SMOTE can improve the accuracy of classifiers for a minority class as it is an over-sampling approach in which the minority class is over-sampled by creating “synthetic” examples rather than by over-sampling with replacement [34]. However, it should be noted that in introducing synthesized examples, there is also a possibility that the application of SMOTE might have introduced an overestimation of the performance metrics [35] and further studies are required. Comparisons between the diagnosis by our SVM model and diagnosis by radiologists are on-going and the potential improvements of the SVM model to lung cancer diagnosis will be presented later.

## Conclusion

Combined contourlet textural features with patient information including demographic parameters and morphological features helped improve the diagnostics accuracy of discerning between benign and malignant solitary pulmonary nodules.

## Supporting Information

Table S1 **Difference of textural features between benign (B) and malignant (M) groups with P value smaller than 7.4e-5.**
(DOCX)
