# Supplementary material for: Contourlet Textual Features: Improving the Diagnosis of Solitary Pulmonary Nodules in Two Dimensional CT Images
Source: PLoS One. 2014 Sep 24;9(9):e108465. doi: 10.1371/journal.pone.0108465 (PMC4177406; doi:10.1371/journal.pone.0108465)
Supplement: Table S1 — Difference of textural features between benign (B) and malignant (M) groups with P value smaller than 7.4e-5. (DOCX) [file pone.0108465.s001.docx]

Table S1 Difference of textural features between benign (B) and malignant(M) groups with *P* value smaller than 7.4e-5

| Variables | Groups | Mean | SD | Median | Q1 | Q3 | Statistic | *P* |
| --- | --- | --- | --- | --- | --- | --- | --- | --- |
| A-1_5_MEAN | B | 10.71 | 1.26 | 10.89 | 10.36 | 11.41 | -6.707 | <1.0e-5 |
|  | M | 10.57 | 1.16 | 10.74 | 10.20 | 11.23 |  |  |
| A-1_2_Standard_Deviation | B | 7.21 | 0.43 | 7.28 | 7.17 | 7.36 | -4.132 | 3.6e-5 |
|  | M | 7.24 | 0.39 | 7.30 | 7.20 | 7.37 |  |  |
| A-1_3_Standard_Deviation | B | 6.91 | 0.45 | 7.00 | 6.80 | 7.13 | -7.416 | <1.0e-5 |
|  | M | 6.98 | 0.35 | 7.05 | 6.89 | 7.16 |  |  |
| A-1_5_Standard_Deviation | B | 6.20 | 0.52 | 6.26 | 5.83 | 6.61 | -7.503 | <1.0e-5 |
|  | M | 6.32 | 0.47 | 6.42 | 5.97 | 6.67 |  |  |
| A-2_1_Standard_Deviation | B | 6.87 | 0.79 | 7.09 | 6.72 | 7.33 | -5.202 | <1.0e-5 |
|  | M | 6.96 | 0.73 | 7.15 | 6.82 | 7.38 |  |  |
| A-2_5_Standard_Deviation | B | 6.58 | 0.43 | 6.56 | 6.29 | 6.91 | -4.403 | 1.1e-5 |
|  | M | 6.64 | 0.41 | 6.61 | 6.34 | 6.96 |  |  |
| A-3_1_Standard_Deviation | B | 6.97 | 0.80 | 7.18 | 6.88 | 7.38 | -4.080 | 4.5e-5 |
|  | M | 7.02 | 0.80 | 7.22 | 6.96 | 7.42 |  |  |
| A-3_2_Standard_Deviation | B | 7.24 | 0.28 | 7.28 | 7.18 | 7.36 | -5.152 | <1.0e-5 |
|  | M | 7.26 | 0.30 | 7.30 | 7.21 | 7.37 |  |  |
| A-3_3_Standard_Deviation | B | 7.08 | 0.34 | 7.13 | 7.01 | 7.23 | -4.434 | <1.0e-5 |
|  | M | 7.11 | 0.26 | 7.15 | 7.03 | 7.25 |  |  |
| A-3_4_Standard_Deviation | B | 7.15 | 0.28 | 7.19 | 7.07 | 7.27 | -5.063 | <1.0e-5 |
|  | M | 7.18 | 0.20 | 7.21 | 7.11 | 7.28 |  |  |
| A-3_6_Standard_Deviation | B | 6.81 | 0.40 | 6.84 | 6.56 | 7.12 | -4.072 | 4.7e-5 |
|  | M | 6.86 | 0.37 | 6.90 | 6.60 | 7.16 |  |  |
| A-5_5_Standard_Deviation | B | 6.58 | 0.39 | 6.62 | 6.37 | 6.85 | -4.191 | 2.8e-5 |
|  | M | 6.63 | 0.34 | 6.65 | 6.43 | 6.87 |  |  |
| A-6_2_Standard_Deviation | B | 7.24 | 0.31 | 7.28 | 7.19 | 7.36 | -3.521 | 4.3e-5 |
|  | M | 7.24 | 0.40 | 7.30 | 7.20 | 7.37 |  |  |
| A-8_4_Standard_Deviation | B | 6.98 | 0.27 | 7.04 | 6.87 | 7.15 | -5.049 | <1.0e-5 |
|  | M | 7.02 | 0.32 | 7.06 | 6.94 | 7.16 |  |  |
| A-8_6_Standard_Deviation | B | 6.26 | 0.44 | 6.28 | 5.95 | 6.59 | -6.088 | <1.0e-5 |
|  | M | 6.34 | 0.38 | 6.38 | 6.07 | 6.62 |  |  |
| A-1_5_Cluster_tendency | B | 21.16 | 2.53 | 21.45 | 20.30 | 22.63 | -7.155 | <1.0e-5 |
|  | M | 20.84 | 2.30 | 21.09 | 19.95 | 22.21 |  |  |
| A-5_5_Cluster_tendency | B | 20.93 | 2.20 | 21.09 | 19.98 | 22.13 | -4.105 | 4.4e-5 |
|  | M | 20.67 | 2.15 | 20.89 | 19.88 | 21.84 |  |  |
| A-8_6_Cluster_tendency | B | 21.07 | 2.59 | 21.40 | 20.38 | 22.48 | -4.081 | 4.5e-5 |
|  | M | 20.96 | 2.20 | 21.27 | 20.32 | 22.12 |  |  |
| A-6_6_Homogeneity | B | 0.68 | 0.10 | 0.66 | 0.61 | 0.73 | -4.058 | 5.0e-5 |
|  | M | 0.67 | 0.10 | 0.65 | 0.60 | 0.72 |  |  |
| A-5_3_Maximum_probability | B | 0.37 | 0.06 | 0.36 | 0.33 | 0.40 | -4.847 | <1.0e-5 |
|  | M | 0.36 | 0.05 | 0.36 | 0.32 | 0.39 |  |  |
| A-5_4_Maximum_probability | B | 0.35 | 0.07 | 0.34 | 0.31 | 0.38 | -4.894 | <1.0e-5 |
|  | M | 0.34 | 0.06 | 0.33 | 0.30 | 0.37 |  |  |
| A-6_4_Maximum_probability | B | 0.41 | 0.09 | 0.39 | 0.35 | 0.47 | -5.036 | <1.0e-5 |
|  | M | 0.40 | 0.09 | 0.38 | 0.34 | 0.45 |  |  |
| A-6_6_Maximum_probability | B | 0.37 | 0.10 | 0.36 | 0.30 | 0.42 | -4.557 | <1.0e-5 |
|  | M | 0.36 | 0.10 | 0.35 | 0.29 | 0.40 |  |  |
| A-7_3_Maximum_probability | B | 0.42 | 0.07 | 0.42 | 0.37 | 0.46 | -5.216 | <1.0e-5 |
|  | M | 0.41 | 0.07 | 0.41 | 0.37 | 0.45 |  |  |
| A-6_6_Energy | B | 0.20 | 0.10 | 0.17 | 0.13 | 0.25 | -4.265 | 2.0e-5 |
|  | M | 0.19 | 0.10 | 0.16 | 0.12 | 0.23 |  |  |
| A-3_5_Inertia | B | 32.70 | 6.58 | 33.72 | 30.47 | 36.61 | -4.852 | <1.0e-5 |
|  | M | 33.40 | 6.81 | 34.57 | 30.83 | 37.65 |  |  |
| A-5_5_Inertia | B | 38.07 | 5.24 | 38.26 | 34.89 | 41.46 | -4.816 | <1.0e-5 |
|  | M | 38.83 | 5.03 | 38.83 | 35.77 | 41.93 |  |  |
| A-5_6_Inertia | B | 38.81 | 5.42 | 38.99 | 35.74 | 42.23 | -4.938 | <1.0e-5 |
|  | M | 39.58 | 4.72 | 39.69 | 36.89 | 42.48 |  |  |
| A-6_6_Inertia | B | 32.42 | 7.30 | 33.70 | 28.95 | 37.18 | -5.029 | <1.0e-5 |
|  | M | 33.35 | 7.00 | 34.64 | 30.52 | 37.82 |  |  |
| A-7_5_Inertia | B | 32.03 | 6.77 | 32.50 | 28.23 | 36.45 | -4.995 | <1.0e-5 |
|  | M | 33.03 | 6.66 | 33.43 | 29.18 | 37.49 |  |  |
| A-7_6_Inertia | B | 32.62 | 8.50 | 34.82 | 29.44 | 38.24 | -5.892 | <1.0e-5 |
|  | M | 33.91 | 8.15 | 35.88 | 31.32 | 39.10 |  |  |
| A-8_6_Inertia | B | 36.94 | 5.26 | 37.18 | 34.52 | 40.03 | -4.665 | <1.0e-5 |
|  | M | 37.73 | 4.67 | 37.87 | 35.08 | 40.48 |  |  |
| A-6_6_Inverse_Difference_Moment | B | 0.65 | 0.11 | 0.63 | 0.58 | 0.71 | -4.070 | 4.7e-5 |
|  | M | 0.64 | 0.11 | 0.62 | 0.56 | 0.70 |  |  |
| A-1_3_Entropy | B | 1.09 | 0.25 | 1.07 | 0.91 | 1.25 | -4.071 | 4.7e-5 |
|  | M | 1.06 | 0.22 | 1.04 | 0.91 | 1.21 |  |  |
| A-6_6_Entropy | B | 1.03 | 0.28 | 1.11 | 0.92 | 1.23 | -4.000 | 6.3e-5 |
|  | M | 1.06 | 0.28 | 1.14 | 0.95 | 1.25 |  |  |
| A-1_5_Correlation | B | 0.51 | 0.11 | 0.52 | 0.45 | 0.58 | -4.725 | <1.0e-5 |
|  | M | 0.52 | 0.10 | 0.53 | 0.46 | 0.59 |  |  |
| A-1_5_Sum-mean | B | 19.17 | 2.53 | 19.46 | 18.31 | 20.64 | -7.155 | <1.0e-5 |
|  | M | 18.84 | 2.30 | 19.10 | 17.96 | 20.22 |  |  |
| A-5_5_Sum-mean | B | 18.94 | 2.20 | 19.09 | 17.98 | 20.14 | -4.105 | 4.0e-5 |
|  | M | 18.68 | 2.15 | 18.90 | 17.89 | 19.85 |  |  |
| A-8_6_Sum-mean | B | 19.08 | 2.59 | 19.41 | 18.39 | 20.49 | -4.081 | 4.5e-5 |
|  | M | 18.97 | 2.20 | 19.27 | 18.32 | 20.13 |  |  |
| A-6_6_Difference-mean | B | 2.88 | 0.72 | 3.06 | 2.59 | 3.37 | -5.047 | <1.0e-5 |
|  | M | 2.97 | 0.72 | 3.13 | 2.71 | 3.44 |  |  |
| A-7_5_Difference-mean | B | 2.71 | 0.62 | 2.76 | 2.36 | 3.12 | -4.099 | 4.1e-5 |
|  | M | 2.79 | 0.62 | 2.83 | 2.42 | 3.21 |  |  |
| A-7_6_Difference-mean | B | 2.94 | 0.87 | 3.19 | 2.63 | 3.52 | -4.234 | 2.3e-5 |
|  | M | 3.04 | 0.84 | 3.26 | 2.77 | 3.60 |  |  |
| A-1_3_Sum-Entropy | B | 3.00 | 0.34 | 3.01 | 2.82 | 3.21 | -4.287 | 1.8e-5 |
|  | M | 2.97 | 0.30 | 2.98 | 2.78 | 3.17 |  |  |

Abbreviation: B, Benign cases; M, Malignant cases.
